# Integrated analysis of potential pathways by which aloe-emodin induces the apoptosis of colon cancer cells

**DOI:** 10.1186/s12935-021-01942-8

**Published:** 2021-04-27

**Authors:** Dongxiao Jiang, Shufei Ding, Zhujun Mao, Liyan You, Yeping Ruan

**Affiliations:** 1 College of Pharmaceutical Sciences, Zhejiang Chinese Medical University, Hangzhou, 310053 People’s Republic of China; 2 Shaoxing Hospital Of Traditional Chinese Medicine, Shaoxing, 312000 People’s Republic of China

**Keywords:** Aloe-emodin, Colon cancer, Network pharmacology, Apoptosis

## Abstract

**Background:**

Colon cancer is a malignant gastrointestinal tumour with high incidence, mortality and metastasis rates worldwide. Aloe-emodin is a monomer compound derived from hydroxyanthraquinone. Aloe-emodin produces a wide range of antitumour effects and is produced by rhubarb, aloe and other herbs. However, the mechanism by which aloe-emodin influences colon cancer is still unclear. We hope these findings will lead to the development of a new therapeutic strategy for the treatment of colon cancer in the clinic.

**Methods:**

We identified the overlapping targets of aloe-emodin and colon cancer and performed protein–protein interaction (PPI), Gene Ontology (GO), and Kyoto Encyclopedia of Genes and Genomes (KEGG) pathway analyses. In addition, we selected apoptosis pathways for experimental verification with cell viability, cell proliferation, caspase-3 activity, DAPI staining, cell cycle and western blotting analyses to evaluate the apoptotic effect of aloe-emodin on colon cancer cells.

**Results:**

The MTT assay and cell colony formation assay showed that aloe-emodin inhibited cell proliferation. DAPI staining confirmed that aloe-emodin induced apoptosis. Aloe-emodin upregulated the protein level of Bax and decreased the expression of Bcl-2, which activates caspase-3 and caspase-9. Furthermore, the protein expression level of cytochrome C increased in a time-dependent manner in the cytoplasm but decreased in a time-dependent manner in the mitochondria.

**Conclusion:**

These results indicate that aloe-emodin may induce the apoptosis of human colon cancer cells through mitochondria-related pathways.

## Background

Colon cancer is among the most common cancers in the world. The global incidence rate of colon cancer ranks third, following that of lung and breast cancer, and the incidence and mortality rates of colon cancer increase each year [1, 2]. However, most colon cancers are caused by dietary habits, obesity, lack of physical exercise, smoking and other unfavourable risk factors [3]. The fact that colon cancer is not apparent until it is in advanced stages causes some difficulties in its diagnosis and treatment [4]. Surgery and chemotherapy are currently the main methods for the treatment of colon cancer in the clinic, but chemotherapy drugs often result in side effects for patients [5]. Therefore, there is a pressing need for medication with few side effects and good curative effects for the treatment of colon cancer to alleviate the suffering of patients. We know that up to 90% of colon cancers can be prevented by dietary changes [6]. Currently, some small-molecule compounds have proven effective for cancer treatment [7–9].

Aloe-emodin is mainly produced by natural plants, such as *Rheum palmatum* L. and *Cassia occidentalis*, and it is a small-molecule hydroxy-anthraquinone, increasing evidence shows that aloe-emodin has a variety of pharmacological activities, especially antitumour activities [10]. Studies have shown that aloe-emodin can induce the apoptosis of cervical cancer cells, and this effect is related to HPV E6, E7 and glucose metabolism [11]. Aloe-emodin enhances the antiproliferative activity of tamoxifen by blocking the Ras/ERK and PI3K/mTOR pathways in breast cancer cells [12]. At present, studies have demonstrated that aloe-emodin has anti-migration and anti-angiogenesis activities in colon cancer cells [13]. In addition, the similar natural active anthraquinone derivative emodin induces cell death by promoting the cell cycle arrest and apoptosis of human LS1034 colon cancer cells in vitro and in vivo [14]. However, the mechanism by which aloe-emodin affects human colon cancer cells is unclear.

It is relatively difficult to accurately illustrate the mechanism of disease management using traditional experimental methods. In recent years, network pharmacology approach would enable an effective mapping of the yet unexplored target space of natural products [15] and it has become a novel and powerful means by which to study the mechanism of action [16]. Network pharmacology also focuses on the prediction of multidrug targets with minimal side effects [17] and combines numerous pharmacological networks with human disease-related genes by emphasizing the multichannel regulation of signalling pathways and revealing disease-related drug targets. Therefore, this approach provides new ideas for the potential mechanism by which aloe-emodin acts in the treatment of colon cancer.

In this study, We used bioinformatics methods to construct protein–protein interaction network [18] by predicting drug and disease targets on five databases (TCMSP, PharmMapper, UniProt, GeneCards, OMIM) were used to identify key targets. The network construction analysis to conduct pathway enrichment analysis and to further explore the mechanism by which aloe-emodin acts in colon cancer. Finally, in vitro experiments were performed to partially verify the conclusions of the network pharmacology analysis, showing significant effect on inducing the apoptosis by which aloe-emodin on human colon cancer cells. The study utilized network based techniques for identifying the potential targets of aloe-emodin in suppressing colon cancer and provide a new theoretical basis for treating colon cancer.

## Materials and methods

### Chemical structure optimization and ADME evaluation

The structural information of aloe-emodin was obtained from PubChem (https://pubchem.ncbi.nlm.nih.gov/, compound CID: 10207). Then, we evaluated aloe-emodin from the Traditional Chinese Medicine Systems Pharmacology (TCMSP) database (https://tcmspw.com/tcmsp.php) through the processes of absorption, distribution, metabolism, and excretion of ADME, and we used two indexes, oral bioavailability (OB) and drug-likeness (DL). OB represents the rate and extent of drug absorption into the blood circulation, and DL describes the molecular properties affecting pharmacodynamics and pharmacokinetics.

### Prediction of potential aloe-emodin targets

The structure of a.sdf file of aloe-emodin (compound CID: 10207) obtained from the PubChem database was optimized by Chem3D 19.0 for the MM2 force field, saved as a mol2 format file and uploaded to PharmMapper (http://www.lilab-ecust.cn/pharmmapper/) for target prediction, where the species was set to human. Results with a z’-score > 0 were selected as targets of aloe-emodin, and the UniProt database (https://www.uniprot.org/) was used to convert the protein names to gene symbols and to remove unverified target genes.

### Colon cancer-associated target prediction

We searched the following two databases for information about the targets of colon cancer. We used colon cancer as a keyword and limited the search to only *Homo sapiens* proteins. The first database was GeneCards (https://www.genecards.org/), a comprehensive and comprehensive collection of known or predicted human genes, and the other database was OMIM (https://omim.org/), which is called the online human Mendelian genetic database in Chinese and passes the scores provided by the database and screens for potential targets.

### Protein–protein interaction (PPI) network construction

First, we identified the targets of aloe-emodin in the two databases and retained only the effective targets related to colon cancer for Wayne’s analysis. We used Cytoscape 3.8.0 software to construct and analyse the networks. Then, we used Genemania (http://genemania.org/) visual network analysis software to analyse the genome in Cytoscape to construct a protein–protein interaction network and predict the aloe-emodin and colon cancer targets. In the PPI protein network, a node represents a protein, and the connection between nodes represents the interaction between proteins. Finally, we selected the top 20 targets of high-node degree as the key targets.

### GO and KEGG pathway enrichment analyses

We used Metascape, a powerful gene function annotation and analysis tool that can quickly help users apply batch gene enrichment analysis websites through bioinformatics methods (https://metascape.org/gp/index.html#/main/step1), to enrich the genes shared by aloe-emodin and diseases. Moreover, we restricted the species selection to "*Homo sapiens*". For these results, p < 0.05 was considered to be significant. Then, a heatmap was plotted with http://www.bioinformatics.com.cn, an online platform for data analysis and visualization.

## Experimental validation

### Cell culture

HCT116 cells (Hunan Fenghui Biotechnology Co., Ltd., Hunan, China) were cultured in Dulbecco's modified Eagle's medium (DMEM)(Gibco, USA), and NCM460 cells (Hunan Fenghui Biotechnology Co., Ltd, Hunan, China) were cultured in Roswell Park Memorial Institute(RPMI 1640)(Gibco, USA).All the cells were containing 10% foetal bovine serum (FBS)(Gibco, USA) and 1% penicillin/streptomycin (Gibco, USA). The cells were all cultured in a cell incubator (Thermo, USA) at 37 °C in a 5% CO_2_ environment.

### Cell viability assay

The MTT assay was used for cell viability assessment. NCM460 and HCT116 cells were seeded in 96-well plates at 5000 cells/well. After incubation for 24 h, the cells were treated with various concentrations of aloe-emodin (AE was provided by Shanghai Yuanye Biological Technology Co., Ltd., Shanghai, China, Lot. Y02M11Y16995, HPLC ≥ 97%) at 24 h, 48 h, or 72 h. Then, 0.5 mg/ml MTT (Sigma, USA) was added and incubated at 37 °C under 5% CO_2_ for 4 h, and then, 100 µl DMSO (Guangdong Guanghua Technology Co., Ltd., Guangdong, China) was added to each well. The absorbance was immediately measured at 490 nm by a microplate reader (Power Wave X340, BioTek, USA), and the cell viability and the percentage of cell viability were calculated according to the following formula: OD value of the treated cells/OD value of the control cell × 100%. By definition, the viability of the control cells from the untreated cultures was defined as 100%.

### Colony formation

The effect on cell proliferation was measured through colony formation experiments. HCT116 cells were evenly spread in a 60-mm cell culture dish at 1 × 10^5^ cells/ml and incubated in complete medium for 24 h, treated with various concentrations of aloe-emodin for 24 h and 48 h, and cultured for 7 days. The cells were stained with crystal violet (Shanghai Aladdin Biochemical Technology Co., Ltd., Shanghai, China) for 15 min and then photographed. The cells were counted with ImageJ 1.51 k software.

### DAPI staining

DAPI staining can be used to observe apoptosis and can penetrate the cell membrane to stain double-stranded DNA in the nucleus in order to label cells. First, HCT116 cells were seeded in 6-well plates at 1 × 10^5^ cells per well. After 24 h of culture, the cells were treated with various concentrations of aloe-emodin for 24 h and 48 h. The morphological changes of the cells were observed under a microscope Then, the cells were fixed with ethanol (Hangzhou Chemical Reagent Company, Hangzhou, China), washed with PBS (Hangzhou Northrend Biotechnology Co., Ltd., Hangzhou, China), stained with 10 mg/ml DAPI (4′,6-diamidino-2-phenylindole, Shanghai Beyotime Biotechnology Co., Ltd., Shanghai, China) for 20 min in the dark, and then photographed by fluorescence microscopy (EVOS FL, USA).

### Caspase-3 activity assay

Caspase-3 is a key enzyme in the process of cell apoptosis. A caspase-3 activity kit (Nanjing Jiancheng Biotechnology Co., Ltd., Nanjing, China) was used to analyse the cultured HCT116 cells, and the caspase-3 activity in the cell lysates was determined according to the protocol provided by the manufacturer. The principle of this assay is to assess changes the sequence-specific polymorphisms of caspase-3 (Ac-DEVD-pNA) coupled with the yellow group pNA. When the substrate is cleaved by caspase-3, the yellow group pNA is released, and then, the caspase-3 activity is measured based on the absorbance at 405 nm by a microplate reader (Power Wave X340, Biotek, USA).

### Cell cycle by flow cytometry

The distribution of cells throughout the cell cycle was assessed by flow cytometry. HCT116 cells were seeded on a 6-well plate and cultured overnight. The HCT116 cells were treated with different concentrations of aloe-emodin for 24 h and 48 h. The adherent cells were digested with trypsin containing EDTA (Gibco, USA), collected in complete DMEM (Gibco, USA), centrifuged, washed with PBS (Hangzhou Northrend Biotechnology Co., Ltd., Hangzhou, China), fixed with ethanol at -20 °C overnight, and treated with PI/RNase (BD Biosciences, San Diego, USA). Then, the cell cycle progression was analysed by a Beckman flow cytometer (Beckman, USA).

### Western blotting

HCT116 cells were seeded in a 6-well plate, treated with different concentrations of aloe-emodin for 24 h, washed with prechilled PBS, lysed in lysis solution (Shanghai Beyotime Biotechnology Co., Ltd., Shanghai, China) on ice for 30 min and then collected. The protein concentration was determined by the BCA method. The proteins were separated by 10% SDS–polyacrylamide gel electrophoresis (SDS-PAGE) and then transferred to PVDF membranes. The membranes were blocked with 5% skim milk (Shanghai Yuanye Biological Technology Co., Ltd. Shanghai, China) and then incubated with primary antibodies overnight at 4 °C. Then, the membranes were washed in TBST and incubated with the appropriate horseradish peroxidase-conjugated secondary antibodies for 2 h at 37 °C. Finally, the immunoreactive bands were visualized by using ECL (Affinity Biosciences, Changzhou, China) and exposed with a ChemiDoc Touch Chemiluminescence gel imaging system (Bio-Rad, USA). Then, ImageJ 1.51 k software was used to qualitatively and quantitatively analyse the protein bands.

### Statistical analysis

All the experimental data were collected from at least three independent experiments, and the results are presented as the mean ± standard deviation (SD). Statistical analysis was performed using IBM SPSS, version 22.0 and with GraphPad Prism 8 software. Statistical analysis involved one-way analysis of variance or two-tailed Student’s t-test; if the results did not conform to a normal distribution, then a nonparametric test was used to perform one-way analysis of variance and multiple comparisons using the LSD method (homogeneity of variance) and Tamhane' T2 (uneven variance). *P* < 0.05 was defined as being statistically significant. The statistical significance between the two different drug concentration groups is as follows: *P < 0.05, **P < 0.01 and ***P < 0.001.

## Results

### Chemical structure optimization and ADME evaluation

**Fig. 1:**
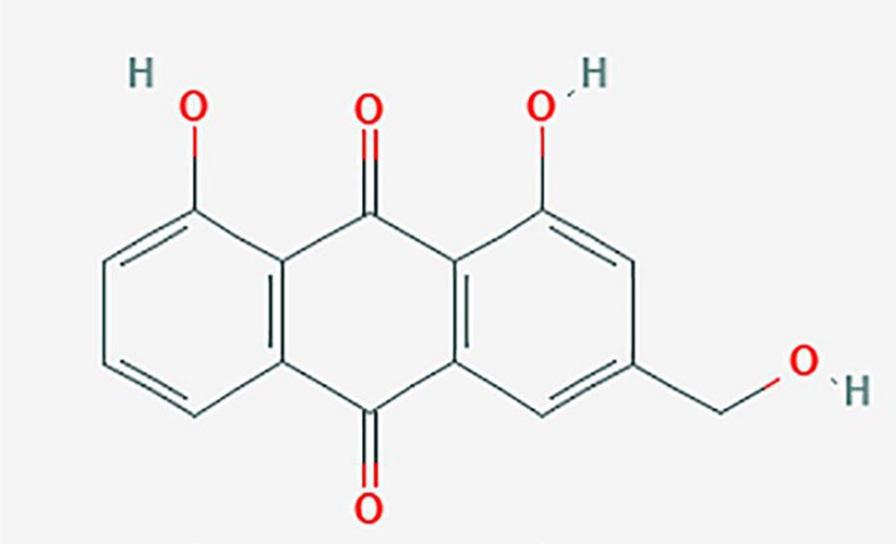
The structural information of Aloe-emodin

The structural information of aloe-emodin was obtained from PubChem (Fig. 1), and the relevant ADME information was obtained from TCMSP. The threshold values ​​of the two indexes are shown in Table 1. This table shows that aloe-emodin has good bioavailability and a good degree of oral utilization.

**Table 1:** OB and DL properties of AE

Name	MW	OB (%)	DL
aloe-emodin	270.25	83.38	0.24

### Target prediction

Based on the potential of aloe-emodin to exert pharmacological effects through multiple targets, we identified its targets through PharmMapper [19] and the UniProt database, which deleted duplicate, unverified targets, and we identified 404 targets of aloe-emodin. In addition, we screened disease targets and identified 845 disease target genes according to the scores provided by the OMIM and Genecards database. To improve the specificity of the targets, we collected the targets of diseases and drugs and identified 43 targets; we selected the overlapping genes for further data analysis.

**Fig. 2:**
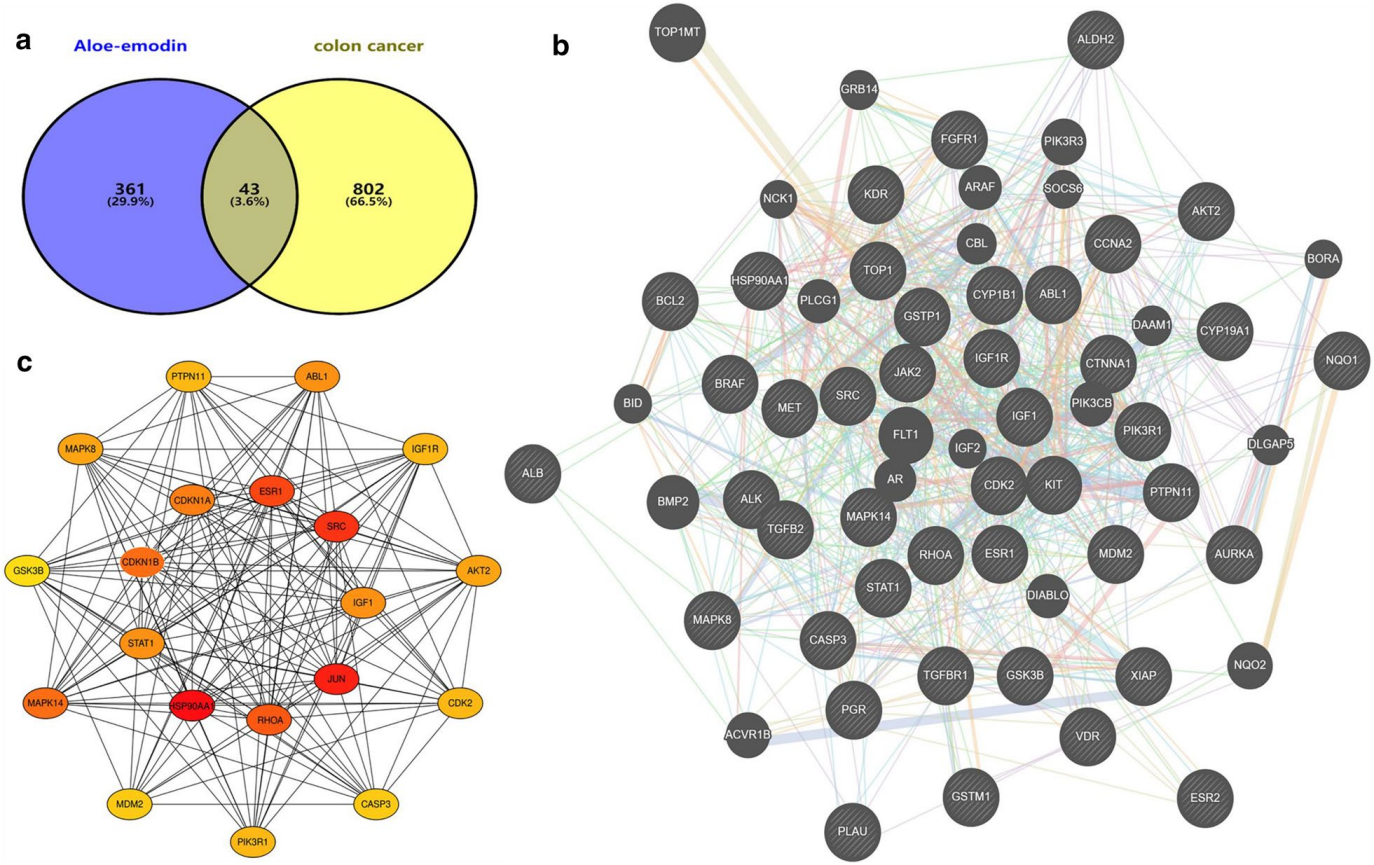
Network construction. **a** Veen diagram of candidate targets of diseases and drugs and obtained 43 collective targets. **b** Protein–protein interaction (PPI) network of of 43 collective targets in aloe-emodin and disease targets. The relationship between the size of circles and degree value of targets is linear positive correlation. **c** Select the top 20 targets from degree value of targets

**Fig. 3:**
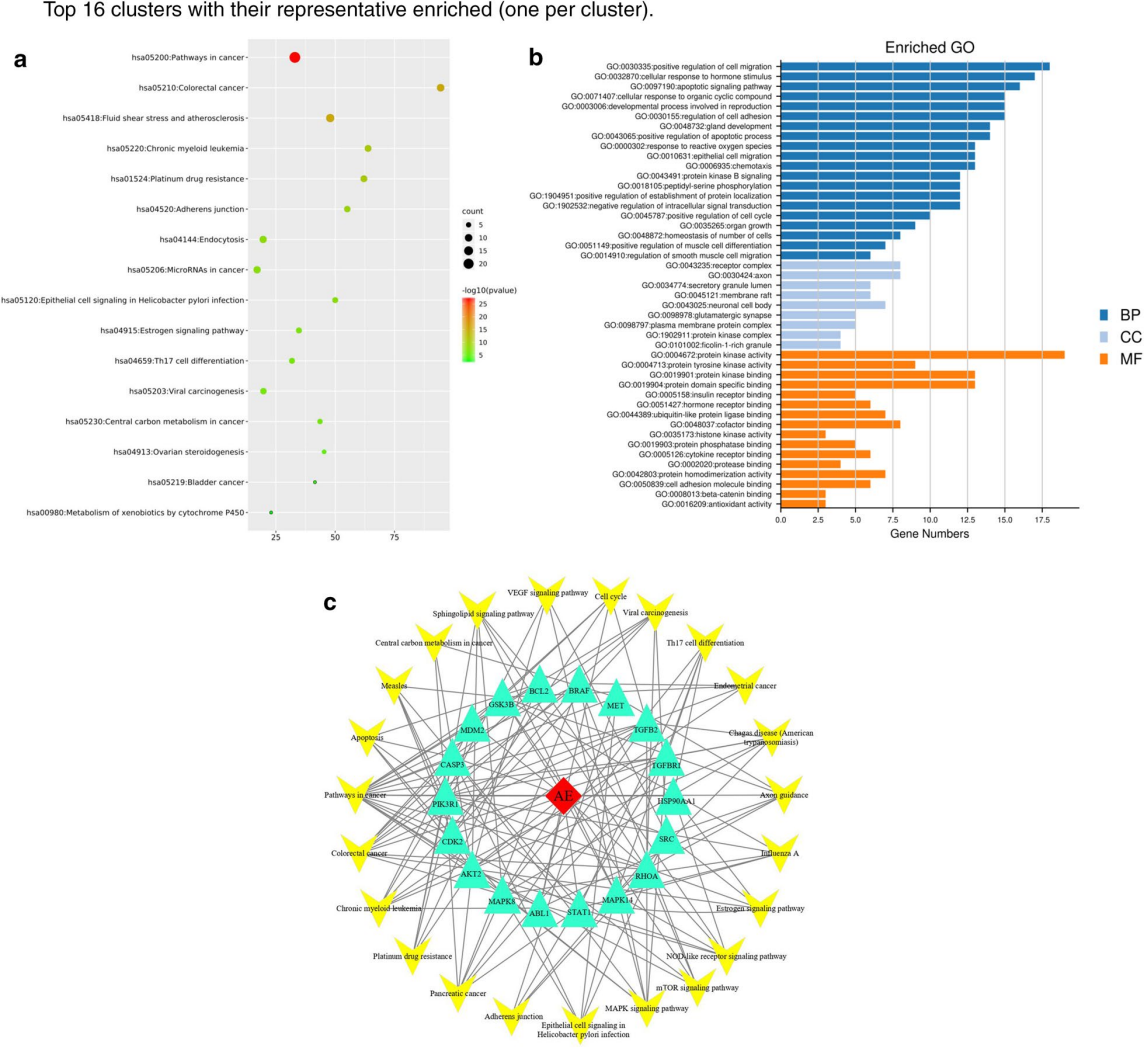
GO and KEGG enrichment analysis. **a** The enrichment analysis of the KEGG signaling pathways. **b** The GO enrichment analysis of potential targets including biological process (BP), cellular component (CC), and molecular function (MF). **c** Construct a target network according to the degree of protein interaction and select protein gene targets with a higher degree of action

**Fig. 4:**
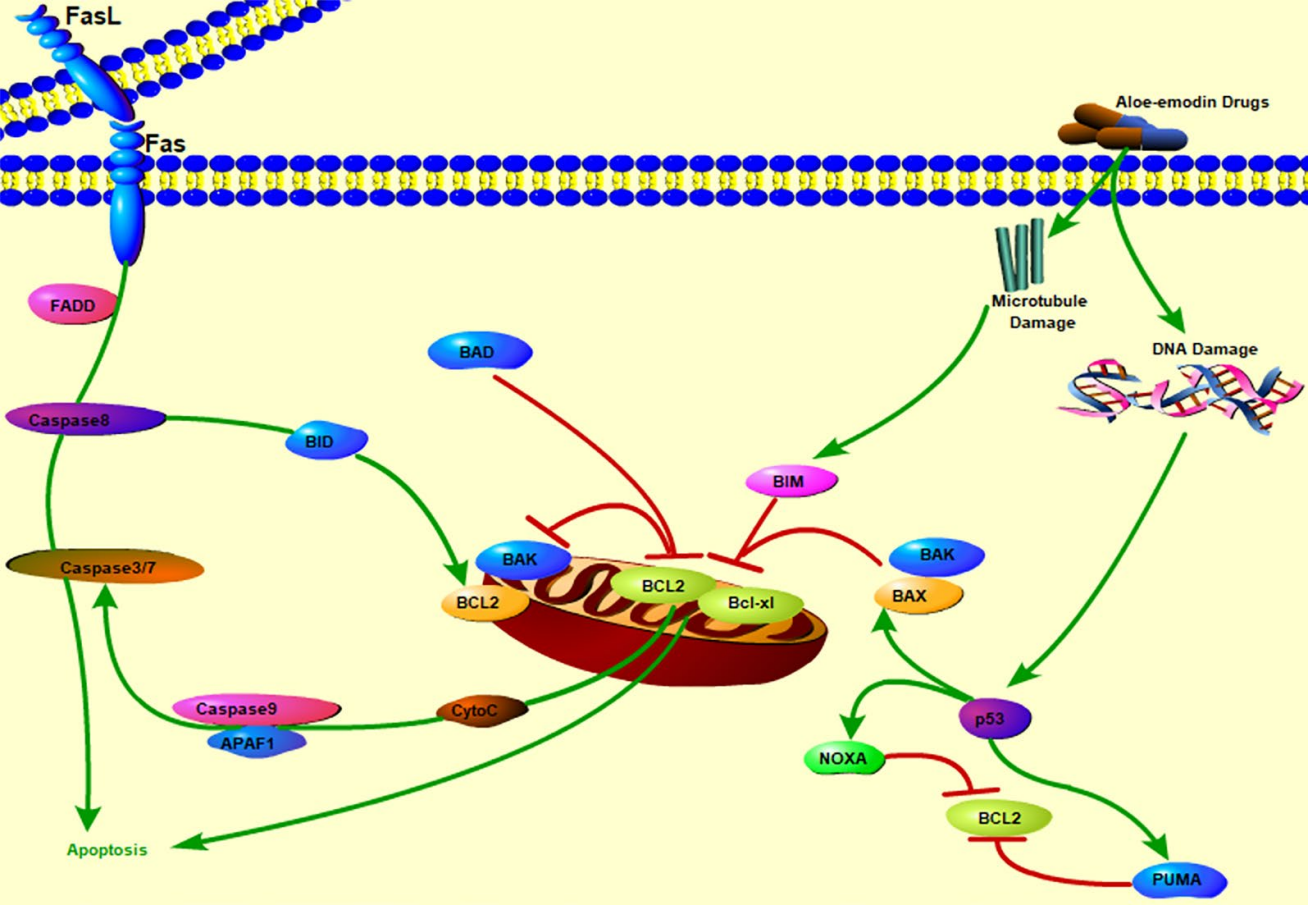
Predict the mechanism of aloe-emodin induced apoptosis of colon cancer

We constructed a PPI network based on 43 targets, and the protein interaction network can simply indicate the interaction between proteins [20]. The result is shown in Fig. 2. The larger the node is, the stronger the degree and the greater the interaction between proteins are, and the straight lines represents the connections between proteins. Then, we used Cytoscape to construct a target network according to the degree of protein interaction and selected protein gene targets with a higher degree of action (Fig. 3c) for KEGG pathway analysis (Fig. 3a) and GO enrichment analysis (Fig. 3b). Among the pathways related to colon cancer, the potential pathways that are significantly related to the aloe-emodin target were identified; these pathways include Pathways in cancer, Colorectal cancer, Proteoglycans in cancer, PI3K-Akt signalling pathway, Prolactin signalling pathway, Neurotrophin signalling pathway, FoxO signalling pathway, and ErbB signalling pathway. The results showed that the Pathways in cancer of the aloe-emodin target had the lowest p-value and the highest overlap. The induction of tumour cell apoptosis plays an important role in the treatment of tumours with drugs. Therefore, we selected the apoptotic pathway in the Pathways in the cancer pathway for experimental verification (Fig. 4).

### Effect of aloe-emodin on the viability of colon cancer cells

**Fig. 5:**
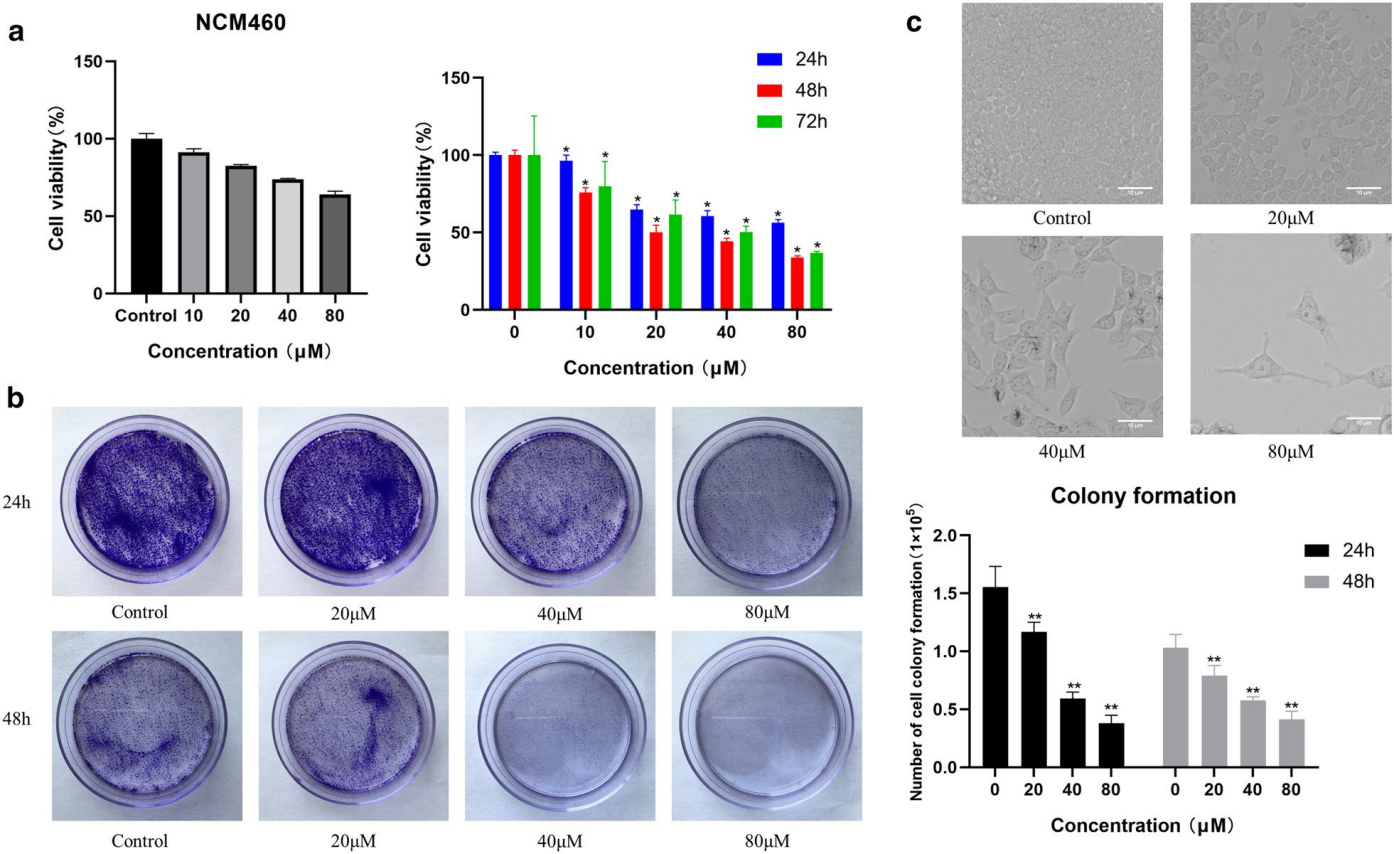
Effect of aloe-emodin on the viability of colon cancer cells. **a** Cell viability was determined by MTT assay after treated with aloe-emodin on NCM460 for 24 h and HCT116 for 24 h, 48 h and 72 h. *P < 0.05 versus Control. **b** Colony formation experiments of HCT116 colon cancer cells treated with aloe-emodin for 24 h, 48 h. **P < 0.01 versus Control. **c** Fluorescence microscope to observe the cell morphology of HCT116 cells treated with aloe-emodin for 48 h (× 20 magnification). All the experimental data were collected from at least three independent experiments. Two-tailed Student’s t-test (**a**), one-way ANOVA (**b**)

MTT assays were used to assess cell viability. First, we measured the effect of aloe-emodin on HCT116 and NCM460 cells. Treating normal colon NCM460 cells with different concentrations of aloe-emodin showed that aloe-emodin had almost no effect or a small effect on the activity of NCM460 which no significant difference in *P* value. In this range, aloe-emodin decreased HCT116 cell viability with increasing dose and time, and the decreasing trend in viability vigour at 48 h and 72 h was consistent (Fig. 5a). In addition, we further demonstrated the antiproliferative effect of aloe-emodin on HCT116 cells through colony formation experiments, and the results were also interesting. The colony formation experiments further showed that when the drug concentration and time increased, the cell mass significantly decreased, indicating that aloe-emodin inhibits cell proliferation in a dose-dependent manner (Fig. 5b). Based on the data results, we used an inverted fluorescence microscope to observe the cell morphology of HCT116 cells treated with aloe-emodin for 48 h, during which time most of the cells lifted off of the culture plate. Compared with untreated normal cells, the apoptotic morphology, such as membrane blistering, increased, and the cells shrank in response to stimulation by drugs (Fig. 5c).

### Effect of aloe-emodin on the apoptosis of colon cancer cells

**Fig. 6:**
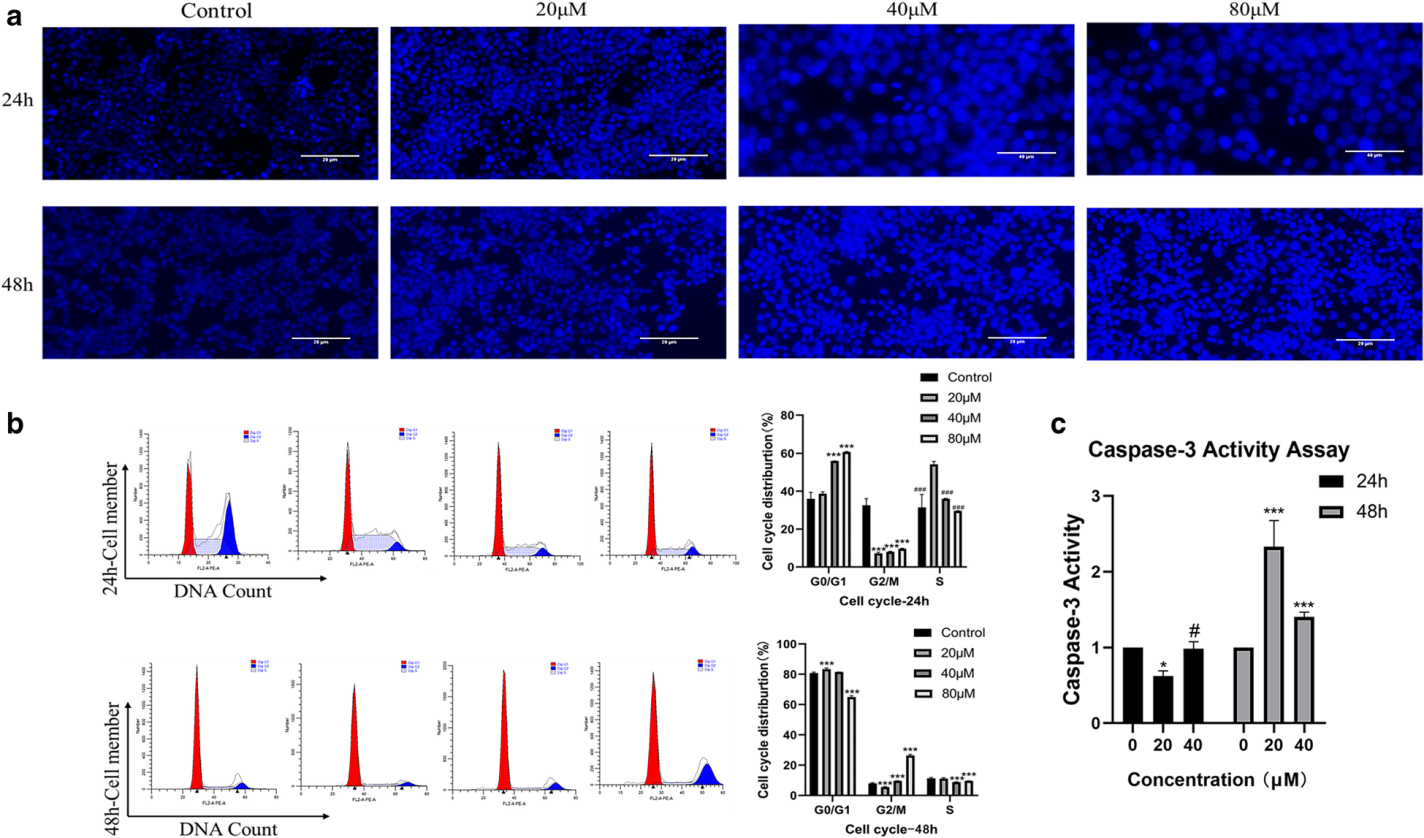
Effect of aloe-emodin on apoptosis of HCT116 colon cancer cells. **a** DAPI staining (× 20 magnification), 40 μM 80 μM for 24 h (× 40 magnification) and the white arrow points to the saient features of apoptotic cells. **b** The cell cycle is assessed by flow cytometry. ***P < 0.001 versus Control. ^###^*P* < 0.001 versus 20 μM. **c** Capase-3 activity assay. Data are expressed as mean ± SD. * *P* < 0.05, ***P < 0.001 versus Control. ^#^*P* < 0.05 versus 20 μM. All the experimental data were collected from at least three independent experiments. Multiple comparisons using LSD method (**b**), Tamhane' T2 and one-way ANOVA (**c**)

According to the above results, we further observed morphological changes in cell apoptosis by DAPI staining (Fig. 6a). Consistent with the results of colon cancer cells treated with drugs, vacuoles appeared in the cytoplasm, and apoptotic bodies appeared. We performed a caspase-3 activity assay to further assess the aloe-emodin-induced apoptosis of HCT116 colon cancer cells. The results showed that the caspase-3 activity in HCT116 cells treated with aloe-emodin for 24 h showed an upward trend, and the caspase-3 activity in the cells the treated for 48 h was significantly higher than that in the cells treated for 24 h (Fig. 6c). Subsequently, we conducted cell cycle measurements and found that the cell arrest induced by aloe-emodin occurred in the G0/G1 phase (Fig. 6b). To further elucidate the mechanism by which aloe-emodin induced apoptosis, we also performed western blot analysis. Compared with those in the control cells, the protein expression levels of Bcl-2, caspase-3, caspase-9 and cytochrome C in the mitochondria in HCT116 cells treated with aloe-emodin decreased, while Bax, cleaved caspase-3, cleaved caspase-9 and cytochrome C in the cytoplasm increased compared with those of the control group. These results suggest that the anti-colon cancer mechanism associated with aloe-emodin may be related to the activation of mitochondrial pathways.

## Discussion

In the past few decades, many studies have reported that natural medicine has many advantages in the treatment of cancer [21, 22]. Aloe-emodin is an anthraquinone compound widely produced by plants, such as *Rheum palmatum L* and *Aloe*, and has significant anti-inflammatory, analgesic, and anti-swelling effects [23]. Aloe-emodin has anti-cell proliferation effects and induces apoptosis in many cancer cells [24, 25], and aloe-emodin can cross HT-29 cellular membranes and pass through the intestinal layer [26]. Therefore, we boldly hypothesize that aloe-emodin is also effective in treating colon cancer. However, few studies have determined the key targets and pathways associated with aloe-emodin in human colon cancer.

Currently, an increasing number of people use biological information technology to study the interaction between human diseases and drug targets and pathways and to predict the biological activities and unknown pharmacological effects of drugs based on screening data, which improves research efficiency and saves time and cost [27]. In this study, for the first time, we used bioinformatics to analyse and predict the potential targets of aloe-emodin and colon cancer and to understand the potential mechanism of drug interaction with the body from a holistic perspective. This study provides new ideas for revealing the mechanism by which aloe-emodin acts the treatment of colon cancer.

In this study, we evaluated the effects of aloe-emodin on the survival and proliferation of colon cancer cells and the possible underlying mechanism. Figure 5 shows that aloe-emodin has little effect on normal colon cells and significantly inhibits the viability of colon cancer cells. In addition, we also found that aloe-emodin significantly inhibited the colony formation of colon cancer cells as the concentration increased, and the cell state gradually showed certain dose-dependent and morphological changes, which were accompanied by changes in membrane blistering and cell needle tip contraction. In addition, DAPI staining showed that in response to the drug, the cells underwent early apoptosis, the nuclear chromatin accumulated in a crescent shape on one side of the nuclear membrane, and the late apoptotic nuclei were fragmented into apoptotic bodies, as shown in Fig. 6.

To explore the functions of aloe-emodin in colon cancer in more detail, we used public databases to conduct network pharmacological analysis to guide future research on aloe-emodin. The results showed that we identified 43 potential targets based on the predicted targeting effect of aloe-emodin on colon cancer through network pharmacology, as shown in Fig. 2. To further elucidate the relevant targets of aloe-emodin in colon cancer, we selected the top 20 relevant targets for analysis; among these targets, HSP90AA1, JUN, SRC, ESR1, RHOA, CDKN1B, MAPK14, CDKN1A, ABL1, MAPK8, AKT2, PTPN11, IGF1R, and PIK3R1 are all related to cell proliferation, and CDK2 plays a key role in cell cycle regulation, and its activity is limited to the G1-S phase of the cell cycle [28]. This is consistent with our experimental results. We analysed the cell cycle by flow cytometry and found that the cells were blocked in the G0/G1 phase, preventing the transformation to the S and M phases, doubling DNA replication and resulting in slow proliferation and reduced cell activity. GO and KEGG pathway analyses showed that aloe-emodin has a wide range of antitumour activities and can regulate a variety of tumour-related pathways. Many factors in cancer development regulate tumour development and may promote or inhibit tumours. Apoptotic cell death serves as an important tumour suppressive mechanism at multiple steps during cancer progression [29–31]. Aloe-emodin can suppress TE1 cell growth by inhibiting AKT and ERK phosphorylation [32]; it induces autophagy through the activation of MAPK signalling and the inhibition of the Akt/mTOR pathway, which triggers apoptosis [33]. In addition, aloe-emodin specifically suppresses breast cancer cell proliferation by targeting ER α protein stability through distinct mechanisms [34], which suggests its value for cancer therapy in the clinic. Through the target-pathway interaction network diagram shown in Fig. 3, we found that these multiple mechanisms of action may be the reason why aloe-emodin protects against different types of cancer.

**Fig. 7:**
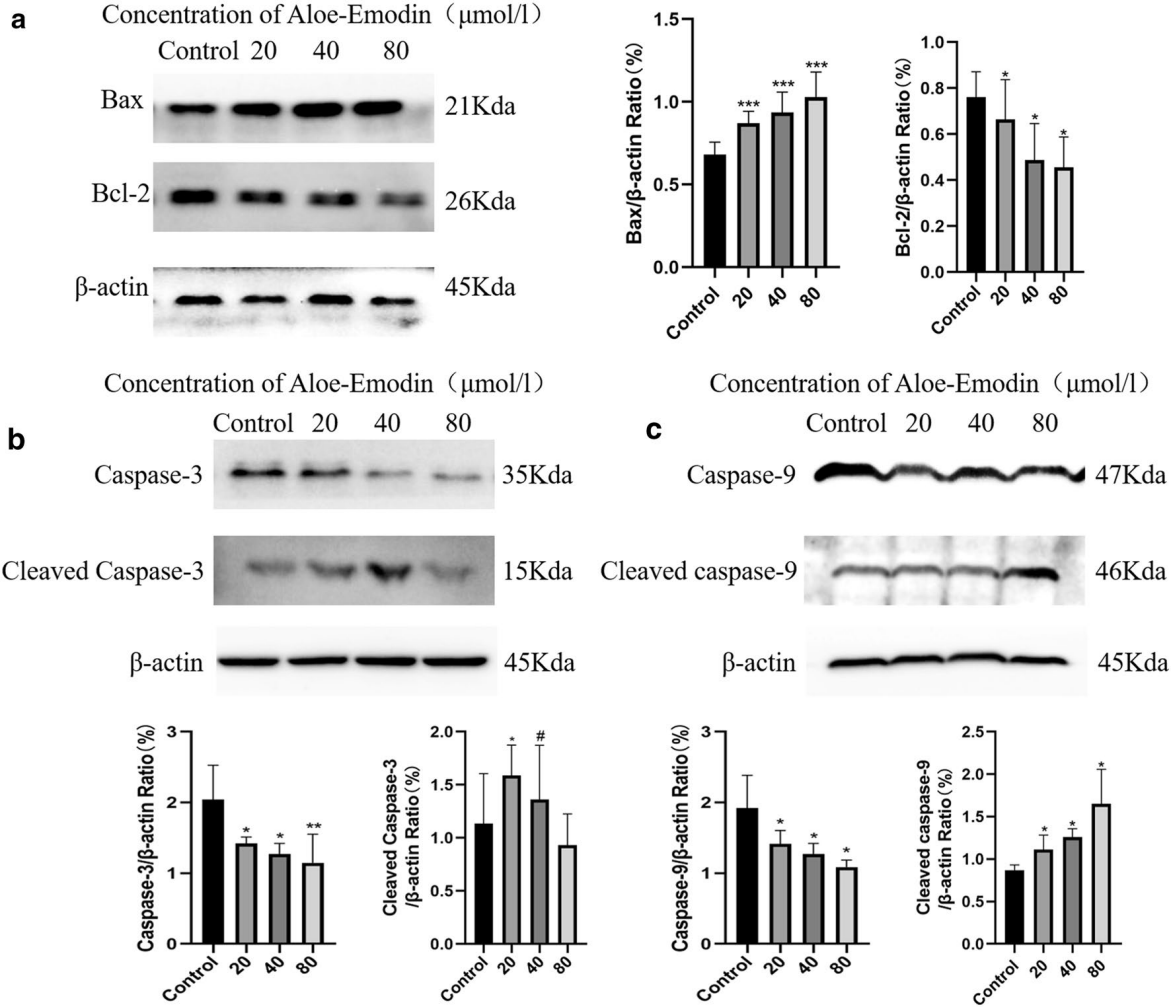
Treatment with aloe-emodin for 24 h and western blotting analysis the protein expression levels. Aloe-emodin activated cleaved-caspase 3, cleaved-caspase 9, and significantly decreased Bcl-2 expression, ultimately causing the release of cytochrome c from the mitochondria into the cytoplasm. All the experimental data were collected from at least three independent experiments. Data are expressed as mean ± SD. *P < 0.05, **P < 0.01, ***P < 0.001 versus Control. ^#^*P* < 0.05, versus 20 μM (Multiple comparisons using LSD method)

**Fig. 8:**
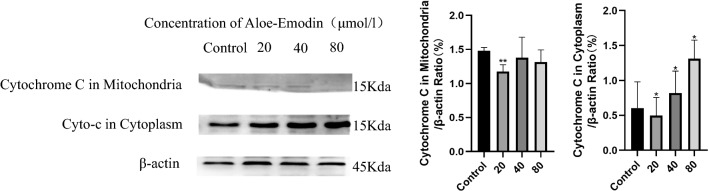
Treatment with aloe-emodin for 24 h, mitochondria and cytoplasm were separated by mitochondrial/cytoplasmic separation kit. Western blotting assessments the expression of Cytochromes C in mitochondria and cytoplasm. All the experimental data were collected from at least three independent experiments. Data are expressed as mean ± SD. *P < 0.05, **P < 0.01 versus Control

Cell apoptosis refers to the autonomous and orderly death of cells to maintain a stable internal environment, and cell apoptosis mechanisms are divided into exogenous pathways and endogenous pathways [35]. The mitochondrial pathway is an endogenous pathway of the cell that is regulated by the interaction between the Bcl-2 family proteins and mitochondria [36]. The Bcl-2 family is divided into proapoptotic factors [37] and antiapoptotic factors. When the mitochondrial pathway is activated, antiapoptotic proteins oligomerize and are inserted into the mitochondria [38], causing the permeability of the mitochondrial membrane to change, the transmembrane potential to be lost and the release of cytochrome C and other proteins into the cytoplasm. In addition, Caspase-3 plays an irreplaceable role in cell apoptosis and is the most important terminal splicing enzyme in cell apoptosis. This study proved that caspase-3 is activated and participates in the cell apoptosis induced by aloe-emodin. Subsequently, we observed the release of the caspase-dependent Bcl-2 family members and cytochrome C during the process of aloe-emodin-induced apoptosis by western blotting, as shown in Figs. 7 and 8. The results showed that the protein expression of the pro-apoptotic Bcl-2 family factor Bax increased while the protein expression of the anti-apoptotic factor Bcl-2 decreased after treatment with aloe-emodin. The pro-apoptotic Bax protein is the main effector of mitochondrial permeabilization during apoptosis [39]. In addition, our results showed that the protein expression level of cytochrome C in the cytoplasm increased in a time-dependent manner but decreased in the mitochondria in a time-dependent manner. The release of cytochrome C is a key step in the mitochondrial apoptosis pathway [40–42]. In addition, the activity of caspase-3 increased, and caspase-9, which may be related to the release of cytochrome C into the cytoplasm, also increased. The release of cytochrome C from the mitochondria to the cytoplasm is the beginning of the mitochondrial apoptosis pathway of cells [43]. Caspase-3 and caspase-9 are executors of and participants in the mitochondrial pathway. Cytochrome C is released from mitochondria and subsequently activates caspases [44]. Therefore, our results provide preliminary evidence that aloe-emodin may induce apoptosis in human colon cancer cells through mitochondria-related pathways, which further suggests the anti-colon cancer effect of aloe-emodin. However, there are some limitations. For example, due to cost and time constraints, this study only verified the key molecules in the mitochondrial apoptosis pathway. Second, some targets with low enrichment scores were not examined in this study. In addition, our findings were verified only in vitro, not in vivo.

## Conclusion

In our current research, we explored the potential targets and mechanisms of aloe-emodin through network pharmacology. The results showed that 43 aloe-emodin targets related to colon cancer were identified, and the top 20 related pathways were extracted from the KEGG database. Aloe-emodin-induced the apoptosis of colon cancer cells may occur by activating the mitochondrial pathway, upregulating caspase-3, caspase-9, and Bax, and downregulating the expression of Bcl-2, which may provide new ideas for future drug development. Nevertheless, the target through which aloe-emodin activates the mitochondrial pathway still needs further exploration.

## Data Availability

All data generated or analyzed during this study are included in the manuscript.

## Abbreviations

AE: Aloe-emodin
OB: oral bioavailability
DL: drug-likeness
PPI: Protein–protein interaction
GO: Gene ontology
KEGG: Kyoto encyclopedia of genes and genomes
DMEM: Dulbecco's Modified Eagle's Medium
RPMI1640: Roswell Park Memorial Institute
FBS: fetal bovine serum
PBS: Phosphate buffer saline
ANOVA: Analysis of variance
